# Toward the Advancement of Soft Pneumatic Rotary Actuators: A Comprehensive Design Review

**DOI:** 10.3390/mi17050608

**Published:** 2026-05-15

**Authors:** Ehsan Kiani Harchegani, Joško Valentinčič

**Affiliations:** Faculty of Mechanical Engineering, University of Ljubljana, Aškerčeva 6, 1000 Ljubljana, Slovenia; ehsan.kiani@fs.uni-lj.si

**Keywords:** soft pneumatic rotary actuators, soft robotics, pneumatic soft actuation, actuator design

## Abstract

The development of robotic systems that can operate safely and adaptively alongside humans requires actuators that combine compliance with reliable performance. Soft pneumatic rotary actuators (SPRAs) have emerged as promising candidates due to their inherent compliance, lightweight design, and capability to generate smooth rotational motion through elastic deformation. However, the diverse designs and performance characteristics of SPRAs make it challenging to identify optimal configurations for specific applications. This review comprehensively surveys current SPRAs, focusing on structural designs, materials, and fabrication methods. While SPRAs offer advantages such as reduced risk of injury and enhanced adaptability, significant challenges remain in optimizing torque output, rotational range, and durability. By comparing existing designs and highlighting open research challenges, this paper aims to guide the advancement of SPRAs, facilitating their integration into safe, effective robotic systems for industrial, medical, and wearable applications.

## 1. Introduction

The pursuit of developing robotic systems that can seamlessly coexist and collaborate with humans has become a central focus in the field of robotics [1,2]. As robots transition from isolated industrial environments to closer interaction with people, the demand for machines that operate safely and adaptively alongside humans has intensified [3]. Although industrial robots have reached a high level of maturity and are integrated widely across manufacturing sectors, human-centered robotic systems still face significant challenges. These challenges primarily stem from the need to ensure safety and adaptability during physical interaction with humans and uncertain environments [4]. At the core of this challenge lies the design of actuation mechanisms that combine high functionality with intrinsic compliance, enabling robots to perform complex tasks while minimizing the risk of injury or damage [5,6]. Actuators, fundamentally responsible for converting energy into motion and force, thus play a pivotal role in shaping the future landscape of soft and adaptive robotics [7,8].

Among the diverse actuation technologies explored in recent years, pneumatic systems have attracted considerable interest due to their intrinsic advantages, including simplicity in design, exceptionally high power-to-weight ratio, and natural compliance, which mitigates rigid impact during interaction [9]. Soft pneumatic actuators, in particular, have gained prominence as they can deliver smooth, flexible, and safe motion, making them ideal for use cases where adaptability and interaction safety outweigh the need for high precision or stiffness like soft robotic grippers to gently handle fragile items (fruits, lab samples) and in wearable assistive devices (soft exosuits) to support human movement safely [10,11,12]. Some research on pneumatic soft actuation has concentrated on linear motion, exemplified by pneumatic artificial muscles [13,14]. Also, there has been an increasing demand for compact, lightweight, and flexible pneumatic soft actuators capable of generating rotary motion [15]; this demand has been driven by applications requiring controlled rotation and sometimes in constrained spaces [16]. For example, minimally invasive surgical tools can use rotary soft actuators to steer and rotate inside narrow anatomical pathways safely. Similarly, compact robotic wrists/joints for small manipulators or in-pipe inspection robots need lightweight rotary motion to turn and orient tools in tight, confined spaces [17]. This demand has stimulated the development of soft pneumatic rotary actuators (SPRAs), which convert internal air pressure into smooth rotational movements using elastic deformation principles [18]. These actuators provide a compelling alternative to conventional electric motors or rigid pneumatic rotary motors, especially in contexts requiring safe torque output and compliant contact mechanics [19].

An SPRA typically consists of one or more elastomeric chambers or networks of chambers enclosed with flexible, stretchable materials. Upon pressurization, these chambers expand and deform, inducing twisting or rotational motion [20]. The performance characteristics, such as torque generation and rotational range, strongly depend on the internal geometry, material properties, chamber layout, and reinforcement patterns that control localized strain distribution [21]. In contrast to rigid pneumatic motors, which rely on discrete mechanical parts and joints, SPRAs achieve motion through continuous elastic deformation [22]. This key difference grants them unique advantages, including enhanced adaptability to external forces [23], improved resilience against mechanical shocks [24], and the elimination of mechanical friction and backlash [25]. These features contribute to smoother, quieter, and safer interactions with both their environment and human users. Consequently, SPRAs are especially promising for robotic joints, wearable exoskeletons, prosthetics, and bioinspired robotic mechanisms where compliance and user safety are paramount [26].

Despite their significant potential, SPRAs face limitations primarily related to the optimization of design parameters [27], selection of suitable materials [28,29], and fabrication techniques [30]. Numerous actuator architectures have emerged, each aiming to improve specific performance aspects such as rotational displacement range, output torque, optimized movement, response speed, and durability [31]. Unfortunately, a comprehensive and systematic comparison of these various designs is still lacking, which presents a barrier to understanding their relative strengths, weaknesses, and application contexts.

This review paper seeks to fill this gap by providing an extensive and detailed overview of SPRAs. Emphasis is placed on cutting-edge structural designs that maximize performance while maintaining compliance and the latest advances in the materials and fabrication methods that enable repeatable and reliable rotational motion. Additionally, the paper covers some modeling approaches essential for predicting actuator behavior, as well as the integration of sensing technologies to enhance feedback and control, to overcome the inherent challenges of the described works. By presenting a new perspective on the state of the art of these actuators, this review aims to highlight ongoing challenges and promising directions, ultimately supporting researchers, designers, and engineers in advancing the field of soft pneumatic rotary actuation and enabling broader adoption of these actuators in real-world soft robotic applications.

To ensure a structured and consistent analysis, the literature considered in this review was selected from major scientific databases, including Web of Science, Scopus, and Google Scholar, using keywords related to soft pneumatic rotary actuation. The focus was placed on studies demonstrating rotary or torsional motion generated by soft pneumatic systems, including both experimental and modeling contributions. The collected works were then systematically organized according to their dominant actuation principles, enabling a consistent comparison of different actuator architectures and the identification of key design trade-offs and research challenges.

To the best of the authors’ knowledge, this is among the first reviews specifically focused on SPRAs, whereas most existing review papers primarily address linear soft actuators or general soft robotic systems. By concentrating on rotary actuation, this work provides a dedicated classification framework based on actuation mechanisms and presents a unified comparative analysis of rotational performance. This perspective enables a clearer understanding of key design trade-offs and highlights emerging directions for the development of soft rotary actuators.

## 2. Different Mechanisms in SPRAs

**Figure 1:**
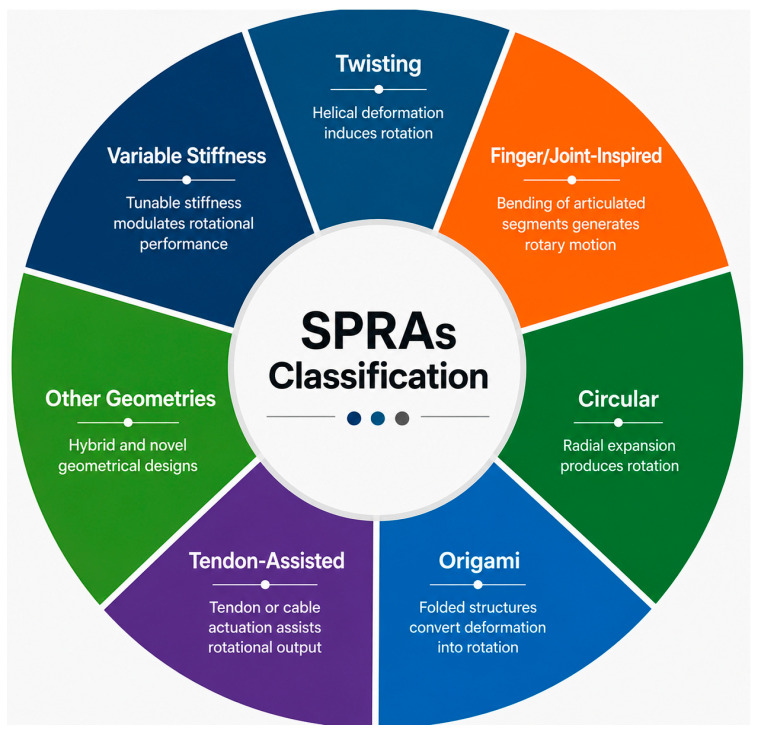
Different design types of SPRAs.

Numerous mechanisms have been developed to generate rotational motion in soft pneumatic rotary actuators (SPRAs). To provide a structured understanding of these approaches, a comprehensive review of the existing literature was conducted. Based on their design architecture and operating principles, while acknowledging that some designs exhibit hybrid characteristics, SPRAs were classified into seven categories, as illustrated in Figure 1. This classification facilitates a clearer interpretation and comparison of the diverse design strategies reported in the literature. Each category represents a distinct mechanical or structural approach for converting pneumatic pressure into controlled rotational output, enabling different performance characteristics such as torque capacity, angular displacement, and response speed. In the following sections, each category is systematically examined in terms of its underlying mechanism, design considerations, and operational behavior, with the aim of highlighting its advantages, limitations, and potential applications in soft robotic systems.

### 2.1. Twisting SPRAs

Twisting SPRAs are a subclass of soft pneumatic rotary actuators designed to generate torsional deformation around their longitudinal axis through pressure-induced shape changes. These actuators are particularly relevant for applications requiring compliant rotational motion, such as joint pronation, object manipulation, and rotational locomotion. As the demand for compact and dexterous soft robotic systems increases, twisting mechanisms have gained significant attention due to their relatively simple structure and ability to produce continuous rotational motion [32].

**Figure 2:**
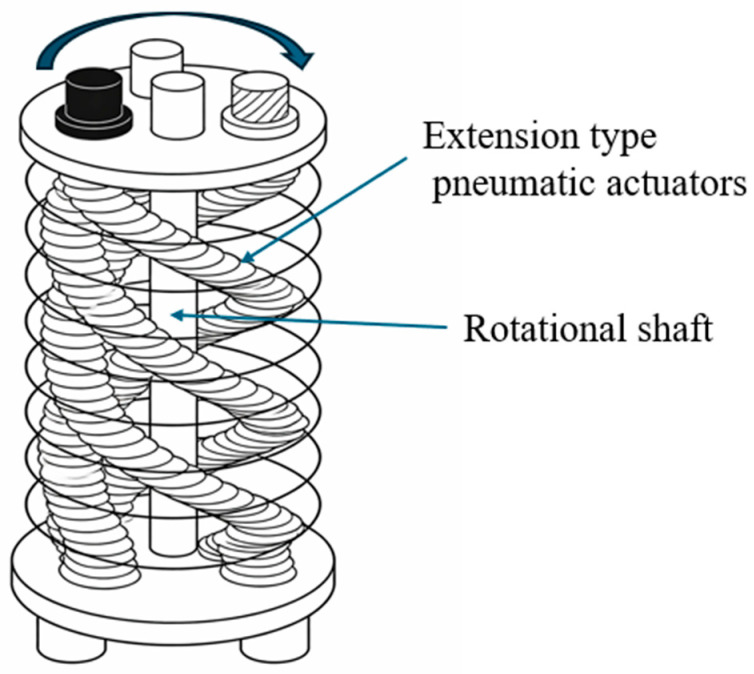
Schematic of a twisting SPRA showing helical extension-type pneumatic actuators arranged around a central shaft. Pressurization induces asymmetric expansion, generating torsional deformation and rotational motion. Adapted from [33].

The fundamental mechanism underlying twisting SPRAs is based on the conversion of isotropic pressure-induced expansion into anisotropic deformation. This is typically achieved by introducing directional constraints within the actuator structure (Figure 2). A widely used approach involves embedding helical or obliquely aligned fiber reinforcements within elastomeric chambers. Under pressure, the natural radial and axial expansion of the chamber is constrained unevenly, resulting in a net torsional deformation [19]. The resulting twist depends strongly on parameters such as fiber angle, chamber geometry, and material stiffness, which together define the balance between axial elongation, radial expansion, and torsional motion.

Several alternative designs have been proposed to enhance twisting performance and expand functionality. Hybrid actuation systems combining pneumatic chambers with double-helical shape-memory alloy wires enable multi-modal actuation, where pneumatic pressure induces bending and extension, while thermal activation of the wires contributes significantly to torsional output [34]. Another approach utilizes pre-twisted pneumatic tubes integrated into soft–hard assemblies, enabling bidirectional rotation and variable stiffness, as demonstrated in forearm-inspired actuators used in multi-degree-of-freedom manipulators [35].

**Figure 3:**
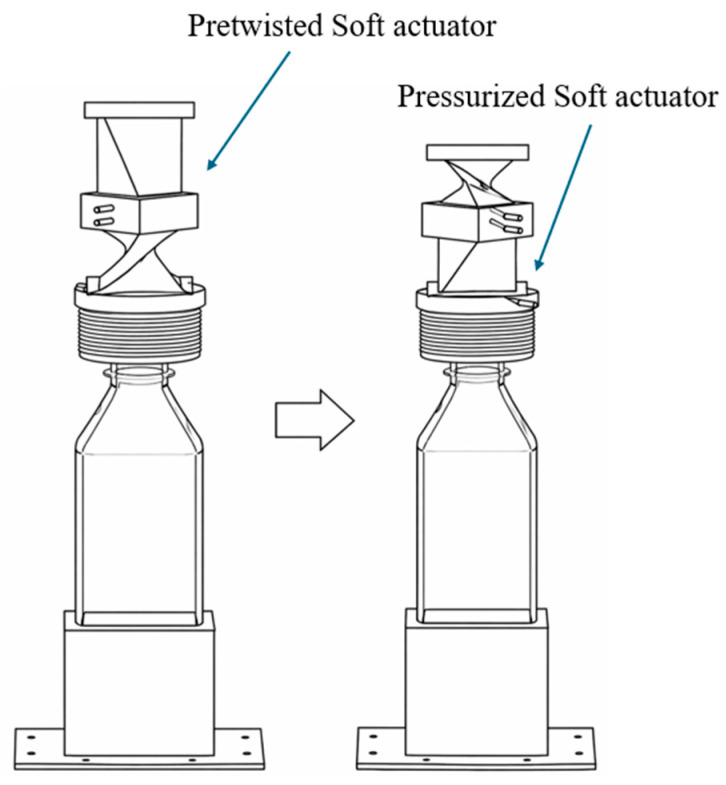
Vacuum-powered twisting actuator enabling multi-mode deformation through controlled chamber collapse under negative pressure. Adapted from [36].

More structurally complex solutions include configurations where multiple extension-type pneumatic actuators are arranged around a central shaft. Coordinated expansion of these actuators generates rotational motion even under non-straight configurations, achieving large rotational ranges of up to 400° and torque outputs of approximately 0.5 Nm at 500 kPa [33]. In contrast, vacuum-driven twisting actuators utilize negative pressure to induce controlled collapse within a seamless chamber (Figure 3), enabling combined linear, radial, and torsional motion without the need for rigid reinforcement [36].

Recent developments have focused on geometry optimization and material-driven design strategies to improve performance. Monolithic freeform chambers optimized through finite element modeling demonstrated bidirectional rotation of up to approximately 116° with torque outputs around 0.8 Nm, while maintaining structural simplicity [37]. Similarly, helical artificial muscle designs based on controlled material collapse have been developed to generate torsion under negative pressure, with surrogate modeling and finite element analysis used to enable inverse design of actuator geometry [38]. Advanced approaches using multi-objective topology optimization have further enabled the design of anisotropic soft joints capable of combining elongation, twisting, and omnidirectional bending within a single structure, expanding their applicability in dexterous robotic systems [39].

Twisting SPRAs provide an effective and relatively compact solution for generating torsional motion in soft robotic systems by exploiting anisotropic constraints on pressure-induced deformation. Their main advantage lies in the ability to produce continuous rotation with relatively simple chamber architecture. However, their performance is fundamentally governed by a trade-off between torsional output and undesired radial expansion. Designs with strong reinforcement, such as fiber-wound chambers, suppress radial deformation and improve torque transmission, but often reduce achievable twist angles and increase stiffness. In contrast, more compliant or minimally constrained designs can achieve larger rotational ranges but typically exhibit lower torque output and reduced motion predictability. In addition, twisting performance is highly sensitive to geometric parameters such as fiber orientation, chamber thickness, and pressure distribution, making systematic design optimization challenging. Hybrid approaches can enhance functionality but introduce additional complexity and potential reliability issues. As a result, twisting SPRAs are well suited for applications requiring moderate torque, compact design, and compliant rotational motion; however, achieving high efficiency, precise control, and long-term durability remains an ongoing challenge. Table 1 summarizes the key performance and structural features of twisting SPRAs reported in the literature.

### 2.2. Finger- and Joint-Inspired SPRAs

Recent advances in soft robotic hands and grippers have focused on improving dexterity and adaptability through the integration of pneumatic actuation, multi-material design, and rigid–flexible hybrid structures. These developments are largely motivated by the need to replicate the complex, multi-axis motion of biological joints such as the human shoulder, elbow, and finger metacarpophalangeal joints [40]. Early soft actuator designs primarily enabled single-axis bending using simple pneumatic chambers, which limited their dexterity and controllability. To overcome these limitations, more advanced architectures have been developed that incorporate additional degrees of freedom and bioinspired structural features [41].

**Figure 4:**
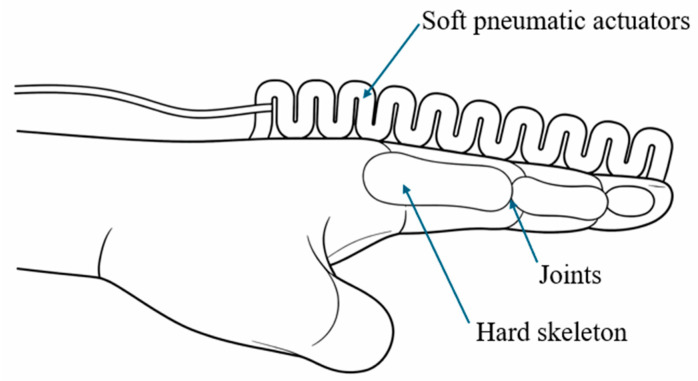
Schematic of finger- and joint-inspired SPRAs, where spatially constrained pneumatic chambers produce controlled bending and rotation that mimic biological joint motion. Adapted from [46].

The underlying actuation mechanism in finger- and joint-inspired SPRAs is based on the non-uniform expansion of elastomeric chambers, which is guided by geometric constraints, segmented structures, and reinforcement strategies (Figure 4). Common design approaches include segmented bellows, fiber-reinforced regions, and constrained inflatable chambers, which allow controlled bending and rotational motion [42,43]. By tailoring the internal chamber geometry and selectively reinforcing specific regions, these actuators can achieve motion patterns that closely resemble biological joints [44]. This capability makes them particularly suitable for tasks requiring safe human interaction and adaptive manipulation [45].

A variety of structural implementations have been explored to improve actuator performance. Multi-material 3D-printed soft finger actuators integrating pneumatic bellows and joint structures have enabled highly dexterous robotic hands with multiple degrees of freedom [47]. Reinforced designs, such as high-modulus silicone actuators combined with aramid fabric layers, have been proposed to reduce lateral expansion and improve force transmission, achieving fingertip forces of approximately 7 N [48]. For wearable systems, detachable pneumatic soft actuators with temporary bonding mechanisms have been introduced to align with biological joints and allow reusability and adaptability [46].

**Figure 5:**
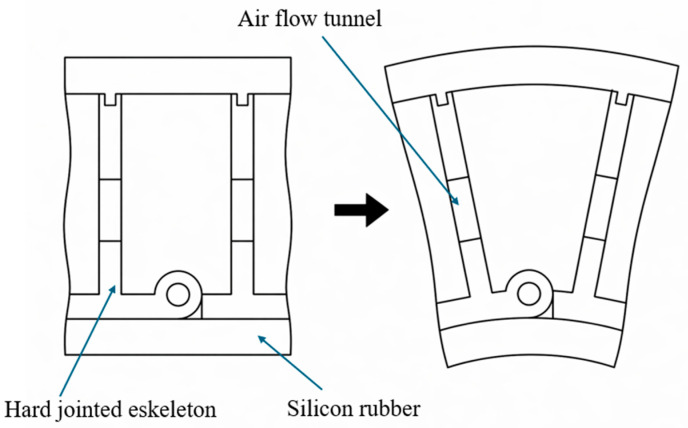
Hybrid soft gripper with a jointed endoskeleton, separating actuation and force transmission to enhance gripping performance. Adapted from [49].

Hybrid architectures combining soft actuation with rigid elements have been widely adopted to improve load capacity and control performance. The E-Gripper (Figure 5) separates actuation from force transmission by integrating pneumatic chambers with a rigid endoskeleton, achieving gripping forces of approximately 35 N at 150 kPa, which is significantly higher than fully soft designs [49]. In addition, finite element modeling combined with vision-based feedback control has been used to improve motion accuracy, enabling soft robotic fingers to achieve controlled joint flexion of up to 90° [50]. Similarly, multi-chamber pneumatic actuators optimized through finite element methods have demonstrated large deformation at low pressure and improved grasp stability through increased contact area [51]. More advanced hybrid systems, such as metacarpophalangeal-inspired grippers using constrained bellows structures, have achieved load capacities exceeding 25 times their own weight while maintaining compliance [52].

Biomimetic principles further extend the capabilities of finger- and joint-inspired SPRAs by enabling more complex and adaptive motion patterns. For instance, a spider-leg-inspired flexible joint actuator employs a double-constrained balloon structure to replicate hydraulic muscle-like behavior, allowing coordinated and compliant motion suitable for soft robotic locomotion systems [53]. Similarly, a frog-inspired miniature soft robot utilizes articulated pneumatic actuators combined with motion planning strategies to achieve efficient swimming and directional control [54]. These examples demonstrate how bioinspired joint architectures can expand the functional range of SPRAs beyond conventional manipulation tasks toward locomotion and environment-specific adaptation.

Further developments have focused on enhancing sensing, adaptability, and bioinspired functionality. A proprioceptive soft actuator combining origami-based structures with embedded sensing elements has enabled simultaneous force and position estimation for improved control [55]. Bioinspired designs, such as a spider-leg-inspired pneumatic gripper, combine rigid linkages, pneumatic joints, and soft contact surfaces to grasp fragile or irregular objects, achieving lifting forces of up to approximately 15 N [56]. Additional designs include hybrid pneumatic rotational actuators with auxetic chambers and bioinspired hinge mechanisms, which aim to improve motion stability and reduce unwanted expansion [57]. Reconfigurable modular systems based on inflatable chambers and structural reinforcements have also been proposed, enabling versatile motion modes such as bending, extension, and locomotion depending on configuration [20]. Finally, hybrid actuation strategies combining tendon-driven mechanisms with pneumatic assistance have been explored to achieve more precise and controllable joint behavior [43].

Finger- and joint-inspired SPRAs provide high levels of motion versatility and biomimetic functionality by combining bending and rotational motion within a single structure. This makes them particularly suitable for soft robotic hands, grippers, and wearable assistive systems. However, these advantages are accompanied by important trade-offs. Increasing the number of joints and degrees of freedom improves dexterity but also increases structural complexity, fabrication difficulty, and control challenges due to coupled nonlinear deformation. Purely soft designs often exhibit limited load-bearing capacity and reduced force transmission efficiency, which motivates the use of hybrid rigid–soft architectures. While these hybrid designs improve strength and precision, they reduce overall compliance and increase system complexity. In addition, multi-material interfaces and segmented structures introduce hysteresis and reduce repeatability over time. As a result, these actuators are most effective in applications where adaptability, safe interaction, and complex motion are prioritized; however, achieving high torque output, precise positioning, and long-term durability remains an ongoing challenge. Table 2 summarizes the key performance and structural features of finger- and joint-inspired SPRAs reported in the literature.

### 2.3. Tendon Assisted SPRAs

Tendon-assisted SPRAs represent an important class of soft pneumatic actuators that combine the compliance of soft materials with the directional force transmission capabilities of tendon-driven systems [58]. This hybrid approach is inspired by biological musculoskeletal systems, where tendons transfer forces from muscles to bones to generate controlled motion across joints [59,60]. By integrating pneumatic actuation with tendon routing, these systems enable more precise control of deformation while maintaining safe interaction with humans and unstructured environments [61].

**Figure 6:**
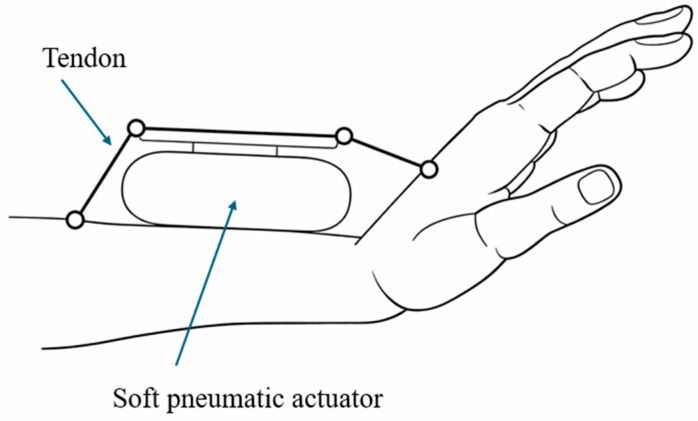
Schematic of a tendon-assisted SPRA, where pneumatic expansion is combined with tendon constraints to guide deformation and enhance force transmission for controlled rotational motion. Adapted from [63].

The core operating principle of tendon-assisted SPRAs relies on the interaction between pressurized elastomeric chambers and inextensible tendons (Figure 6). Upon pressurization, the soft actuator undergoes deformation, while the tendons guide or constrain this motion to produce controlled bending or rotation. Tendons can either assist deformation by redirecting forces or resist it to shape the actuator response. In many designs, antagonistic tendon configurations are used, allowing bidirectional motion and adjustable stiffness through coordinated tension and pressure control [62]. This coupling between pneumatic expansion and tendon mechanics enables improved torque generation and motion precision compared to purely soft actuators [63].

Early tendon-driven robotic systems primarily relied on rigid structures, which provided efficient force transmission but lacked adaptability and safety during interaction [64]. The integration of soft pneumatic elements introduced a new paradigm, combining compliance with controlled actuation. As a result, tendon-assisted SPRAs can achieve high force output, tunable stiffness, and compact form factors, making them particularly suitable for wearable and assistive applications [65].

A variety of implementations have been developed across different application domains. In rehabilitation robotics, a soft wearable upper-limb system has been designed to provide controlled joint motion while improving comfort compared to rigid exoskeletons, supported by both experimental validation and kinematic modeling [63]. Similarly, a multi-joint exosuit combining tendon-driven hip actuation with a pneumatic knee module demonstrated reduced metabolic cost and muscle activity during assisted walking, while preserving natural gait patterns [66].

**Figure 7:**
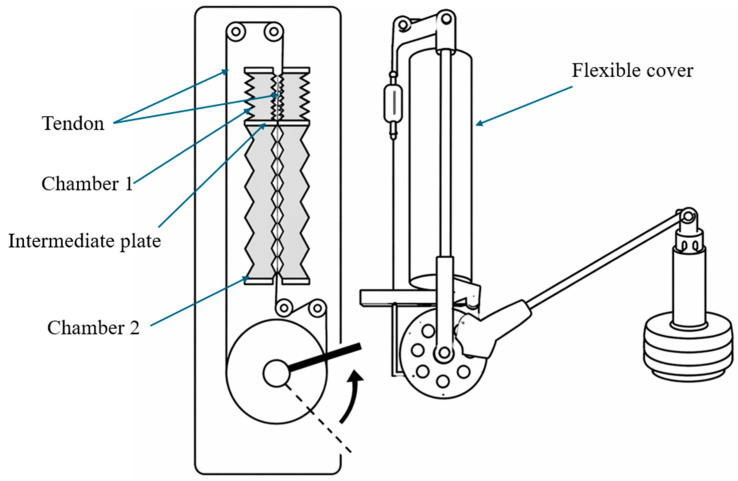
Schematic of a bidirectional pneumatic rotary actuator illustrating tendon-driven transmission and dual-chamber actuation. Adapted from [67].

To enhance force output and bidirectional control, antagonistic tendon-driven actuators have been developed (Figure 7), capable of generating contraction forces of approximately 124 N and expansion forces of approximately 123 N. When integrated into robotic arms, such systems have demonstrated the ability to lift loads of up to 2 kg over large angular ranges [67]. In the field of surgical robotics, a compact soft pneumatic rotary actuator combined with a cam mechanism has been proposed for laparoscopic applications, achieving rotational motion exceeding 70° with torque output suitable for delicate manipulation tasks such as needle insertion [68].

Wearable pneumatic systems that employ tendon-assisted actuation typically rely on compact pressure sources, such as miniature compressors or CO_2_ cartridges, combined with valves and sensors to regulate pressure and ensure safe operation [63,69,70]. These systems are often designed to be untethered, with portable air supply units integrated into wearable platforms, enabling practical deployment outside laboratory environments.

Tendon-assisted SPRAs offer a compelling balance between compliance and controllability by combining soft pneumatic deformation with tendon-based force transmission. This enables improved torque output, directional control, and bidirectional actuation compared to purely soft designs. However, these advantages introduce important trade-offs. The addition of tendons increases mechanical complexity and introduces friction, wear, and routing constraints, which can reduce efficiency and long-term reliability. Furthermore, the interaction between tendon tension and pneumatic pressure leads to strongly nonlinear and coupled behavior, making modeling and control more challenging. While antagonistic configurations allow stiffness modulation and improved motion control, they also require more sophisticated coordination strategies. As a result, tendon-assisted SPRAs are particularly well suited for applications requiring precise motion and higher force output, such as rehabilitation devices and surgical tools, but they involve increased design and control complexity compared to simpler soft actuator architectures.

Table 3 summarizes the key performance and structural features of tendon-assisted SPRAs reported in the literature.

### 2.4. Variable-Stiffness SPRAs

Variable-stiffness actuation has emerged as a key concept in soft robotics, inspired by biological systems where muscles regulate joint stiffness to achieve both adaptability and load-bearing capability [71]. In the context of SPRAs, variable-stiffness mechanisms aim to dynamically adjust the mechanical response of the actuator, allowing transitions between compliant and stiff states depending on task requirements. This capability is essential for applications involving physical interaction with uncertain environments, where both safety and performance must be balanced.

**Figure 8:**
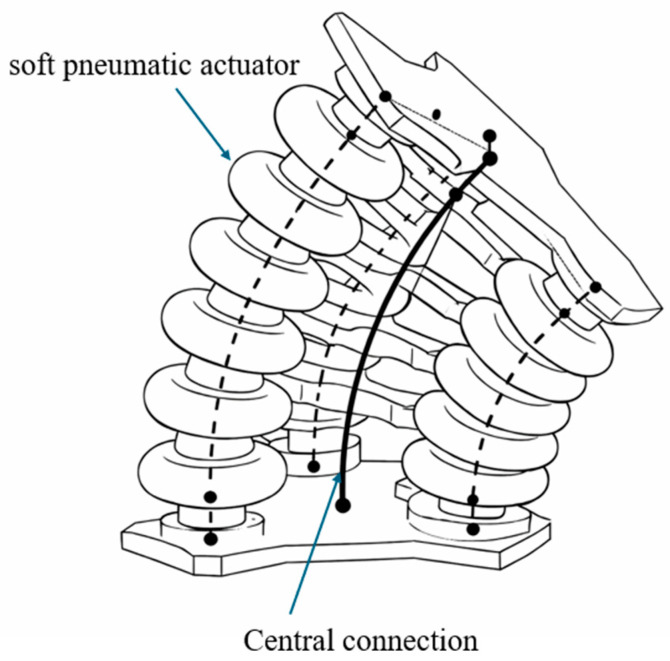
Schematic of a variable-stiffness SPRA illustrating stiffness modulation through antagonistic actuation, structural reinforcement, or hybrid soft–rigid elements. Adapted from [72].

The underlying principle of variable stiffness in SPRAs is based on modulating the effective structural resistance of the actuator during operation. This can be achieved through several mechanisms, including antagonistic actuation, geometric reconfiguration, and structural reinforcement. In antagonistic configurations, pairs of opposing actuators are simultaneously pressurized, creating internal preload that increases joint stiffness while maintaining compliance (Figure 8) [72]. This approach mimics biological muscle co-contraction and enables continuous tuning of stiffness without requiring additional mechanical components.

Another important strategy involves structural reinforcement and geometric modulation, where stiffness is altered by changing the actuator configuration or internal structure. For example, soft continuum manipulators inspired by biological systems such as squid tentacles and elephant trunks integrate molded silicone actuators with embedded wave springs, achieving up to a tenfold increase in torsional stiffness while maintaining flexibility [72]. Similarly, hybrid systems combining pneumatic actuation with cable-driven mechanisms have been developed for applications such as assisting elderly individuals, where both adaptability and controlled force transmission are required [71].

Recent developments have explored more advanced approaches to stiffness modulation based on modular design and structural layering concepts. In these systems, stiffness can be adjusted by selectively activating or reconfiguring structural elements, enabling localized control of deformation. For instance, soft robotic joints combining rigid vertebra-like elements with antagonistic origami-based actuators have demonstrated multi-degree-of-freedom motion with improved load-bearing capacity and torque output of approximately 1.7 Nm [73]. Modular systems incorporating spring-reinforced soft actuators, tunable stiffness pads, and embedded sensing elements have further enabled real-time stiffness adjustment and improved motion control through feedback integration [74].

In addition to geometric and modular approaches, material-driven stiffness modulation has been explored through the use of composite structures and functional materials. Soft robotic arms integrating multiple sensing and actuation modalities, including inertial sensing and touch-responsive elements, have demonstrated high positioning accuracy with errors below 1 mm and improved durability over repeated cycles [75]. Similarly, multi-actuator configurations such as parallel helical actuator systems have been designed to provide both bending and twisting motion with adjustable stiffness, supported by finite element modeling and kinematic analysis [75].

**Figure 9:**
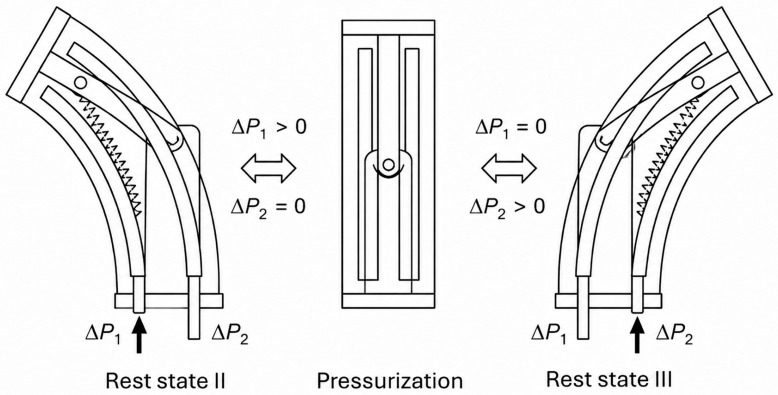
Schematic illustration of the bistable working mechanism during pressurization. The structure transitions between two stable configurations, Rest state II and Rest state III, driven by differential pressure inputs (ΔP_1_ and ΔP_2_). When ΔP_1_ > 0 and ΔP_2_ = 0, the system is driven toward one stable state, whereas reversing the pressure condition (ΔP_1_ = 0, ΔP_2_ > 0) induces a transition to the alternate state via reversible snap-through. The central panel illustrates the pressurization configuration controlling the bistable switching. Adapted from [76].

A distinct class of variable-stiffness mechanisms is based on mechanical instability and bistability, where stiffness is controlled through rapid transitions between stable states. For example, systems exploiting snap-through bistability can store and release energy efficiently, enabling significant stiffness variation without continuous energy input. Such designs have demonstrated stiffness modulation of up to three orders of magnitude, along with the ability to support loads exceeding 10 kg in applications such as crawling robots, swimmers, and adaptive grippers (Figure 9) [76]. Additionally, biologically inspired systems, such as musculoskeletal shoulder modules with multiple independently actuated pneumatic muscle bundles, have achieved large motion ranges exceeding 180° while maintaining high energy density [77].

Variable-stiffness SPRAs address one of the central challenges in soft robotics, namely the trade-off between compliance and load-bearing capability. By enabling dynamic adjustment of stiffness, these actuators can adapt to different task requirements, providing both safe interaction and improved mechanical performance. However, this adaptability introduces significant trade-offs. Increasing stiffness typically requires additional actuation, structural elements, or control complexity, which can reduce system efficiency and increase design difficulty. Antagonistic actuation provides continuous stiffness tuning but demands precise coordination and higher energy consumption. Structural and modular approaches improve load capacity but may reduce flexibility and increase fabrication complexity. Material-based strategies and bistable mechanisms offer energy-efficient stiffness modulation, but they often introduce limitations in controllability or repeatability. Furthermore, integrating sensing and control for real-time stiffness adjustment remains a major challenge due to nonlinear and coupled system behavior. As a result, variable-stiffness SPRAs are highly promising for applications requiring adaptive interaction and load handling, but further advances are needed in simplified design, energy efficiency, and robust control strategies to fully realize their potential. Table 4 summarizes the key performance and structural features of variable-stiffness SPRAs reported in the literature.

### 2.5. Circular SPRAs

Circular SPRAs are designed to generate controlled rotational motion through the coordinated actuation of multiple pneumatic chambers arranged around a central axis [79]. Unlike twisting or joint-inspired designs that rely on localized deformation, circular SPRAs aim to achieve servo-like rotary motion by distributing actuation symmetrically, making them conceptually closer to conventional rotary actuators while retaining the compliance of soft systems [80].

**Figure 10:**
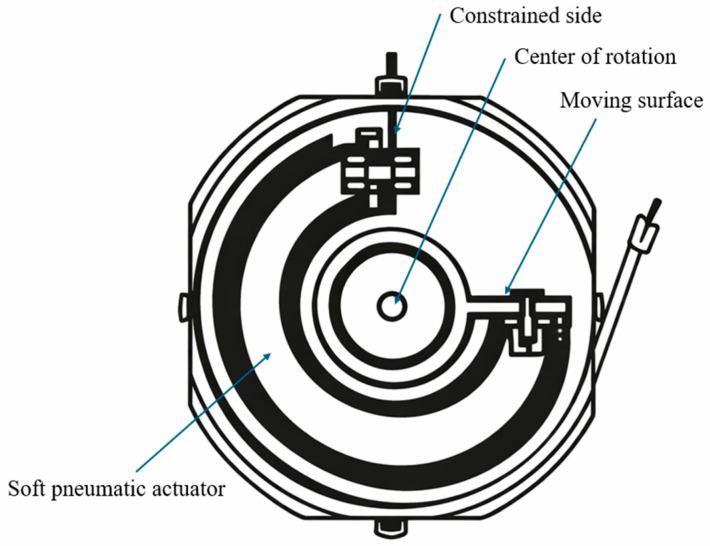
Schematic of a circular SPRA, where differential pressurization of circumferential chambers generates bidirectional rotational motion about a central axis. Adapted from [15].

The fundamental operating principle of circular SPRAs is based on differential pressurization of circumferentially arranged chambers (Figure 10). By selectively inflating and deflating these chambers, a net rotational moment is generated around a central shaft. In many designs, this is achieved through antagonistic actuator pairs, where one chamber is pressurized while the opposing chamber is depressurized, producing bidirectional rotation [15]. The resulting motion depends on the balance of internal pressures, chamber geometry, and mechanical constraints imposed by the central structure.

Early implementations focused on simple configurations using arrays of pneumatic chambers arranged in a ring, enabling cyclic or stepwise rotational motion [80]. More advanced designs have introduced antagonistic bidirectional systems, where paired silicone actuators are mounted around a central joint. These systems can achieve controlled rotation within a defined angular range by coordinating pressure inputs, allowing rotation between approximately 0° and 90° in one direction and up to about −16° in the opposite direction [80].

To improve controllability and load-bearing capability, additional mechanisms such as pneumatic braking systems have been integrated into circular SPRAs. For example, an antagonistic rotary joint combining semicircular actuators with a braking mechanism has been proposed to enable fast motion, accurate position holding, and increased resistance to external loads [15]. In wearable applications, circular actuation has been applied to assist thumb motion in soft robotic gloves, where both single-actuator and dual-actuator configurations have been evaluated. The dual-actuator design demonstrated improved force output and abduction control, while the single-actuator configuration offered better coordination and functional performance [81].

These systems are often integrated with external sensing and control interfaces to enhance usability. For instance, gesture-controlled circular SPRAs have been developed using motion-tracking systems, enabling real-time control of actuator rotation through user inputs such as hand gestures [80]. This highlights their potential for intuitive human–robot interaction and assistive applications.

Circular SPRAs provide a structured approach to achieving controlled rotary motion by distributing actuation around a central axis, making them conceptually closer to traditional rotary actuators than other soft actuator designs. Their main advantage lies in their ability to deliver smooth, repeatable, and controllable rotational motion, particularly when combined with antagonistic actuation and external control systems. However, this approach introduces several important trade-offs. The reliance on multiple chambers and coordinated pressure control increases system complexity and requires precise synchronization to achieve accurate positioning. In addition, circular SPRAs typically exhibit limited torque output and restricted rotational range compared to twisting or tendon-assisted designs, especially when constrained by compact geometries. Increasing the number of chambers can improve motion smoothness and control resolution, but also increases fabrication complexity and system size. Furthermore, maintaining a stable position under load often requires additional mechanisms such as brakes or continuous pressure input, which can reduce energy efficiency. As a result, circular SPRAs are particularly suitable for applications requiring controlled, low-to-moderate torque rotation and intuitive human interaction, such as wearable devices and assistive systems, while their use in high-load or high-torque applications remains limited. Table 5 summarizes the key performance and structural features of circular SPRAs reported in the literature.

### 2.6. Origami-Inspired SPRAs

Origami-inspired soft actuators represent an advanced design approach in soft robotics, where geometric folding principles are used to achieve complex and programmable deformation. By integrating foldable patterns into soft structures, these actuators can generate rotational, bending, and multi-degree-of-freedom motion while maintaining a compact and lightweight form. Their ability to encode motion directly into geometry makes them particularly attractive for applications requiring reconfigurability and high functional density [82,83].

**Figure 11:**
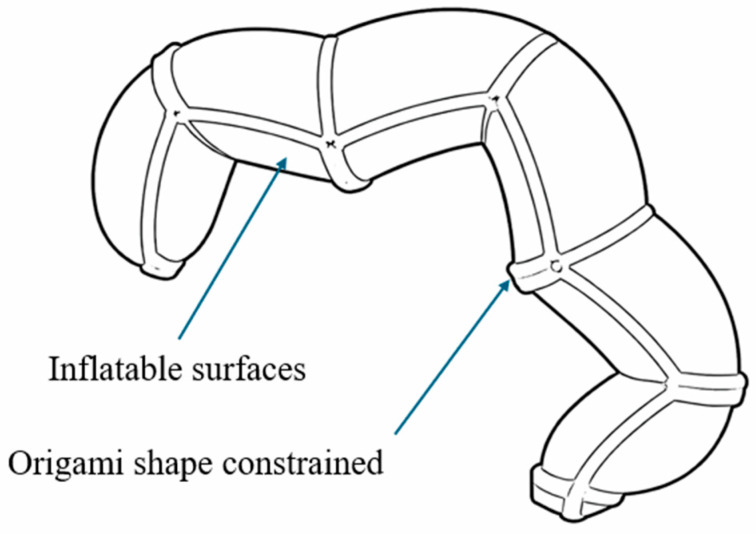
Schematic illustration of an origami-inspired soft pneumatic rotary actuator, showing deformation through folding-induced bending and rotation. Adapted from [90].

The operating principle of origami-inspired SPRAs is based on the interaction between geometric folding constraints and pressure-driven deformation. Unlike conventional soft actuators that rely primarily on material elasticity, origami-based designs utilize predefined fold patterns such as Kresling, Miura-ori, and waterbomb structures to guide deformation in a predictable manner [84,85,86]. When pressurized, these folded structures undergo coordinated expansion and folding transitions, converting internal pressure into controlled rotational or multi-axis motion (Figure 11) [87]. This combination of soft materials and geometric constraints enables large deformation ranges while maintaining structural integrity [88,89].

The use of origami principles in engineering originated from deployable structures in aerospace and medical devices, where compact-to-expanded transformation is essential [91]. In soft robotics, these principles have been adapted to create actuators capable of complex motion with minimal actuation input, often achieving multiple functions within a single structure. For example, bioinspired origami-based systems have enabled multifunctional robots, such as a dual-morphing actuator inspired by the pelican eel, which combines folding and inflation to achieve gripping, crawling, and swimming behaviors [90].

**Figure 12:**
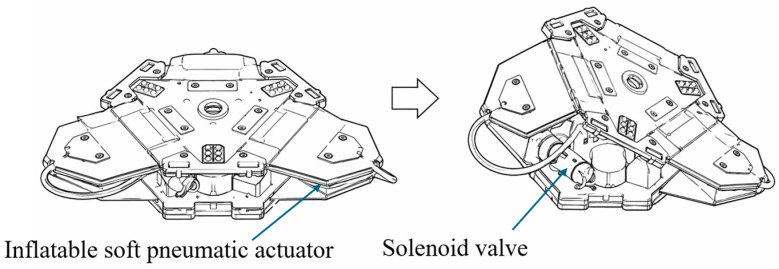
Modular origami-inspired spherical joint enabling decentralized pneumatic control and coordinated motion. Adapted from [92].

To address scalability and control, modular origami architectures have been developed. A modular platform based on origami-inspired spherical joints (Figure 12) demonstrated decentralized control using passive pneumatic relays, enabling coordinated motion with reduced control complexity [92]. In addition, 3D-printed origami rotary actuators have achieved torque outputs of up to approximately 18.5 Nm at 180 kPa while incorporating impact-safety features that reduce torque during collisions, improving safety and robustness [93].

Origami-inspired SPRAs have also been applied in assistive and wearable systems. For example, origami-shaped pneumatic actuators integrated into wrist support devices have enabled controlled bending and lateral motion for rehabilitation applications [94]. Furthermore, origami-based pneumatic joints capable of operating under both positive and negative pressure have demonstrated bidirectional rotation, enabling multi-degree-of-freedom manipulators capable of performing pick-and-place tasks with loads exceeding 1 kg [95].

Origami-inspired SPRAs offer a unique advantage by embedding motion functionality directly into geometric design, enabling highly programmable and multifunctional actuation within compact structures. This approach allows large deformation ranges and complex motion patterns that are difficult to achieve with conventional soft actuator architectures. However, these benefits are associated with several important trade-offs. The performance of origami-based actuators is highly sensitive to fabrication accuracy and material consistency, as small deviations in fold geometry can significantly affect motion behavior. In addition, while thin folded structures enable large deformation, they often exhibit reduced durability and increased susceptibility to fatigue under repeated loading. Another challenge is the sealing and structural stability of fold interfaces, which can limit long-term reliability, especially under high pressure. Although origami geometries can reduce the need for complex control by encoding motion into structure, they may also introduce limitations in controllability and adaptability compared to actively controlled systems. As a result, origami-inspired SPRAs are particularly suitable for applications requiring compact, lightweight, and multifunctional actuation, while further advances in materials, fabrication techniques, and structural robustness are needed to fully exploit their potential in demanding environments. Table 6 summarizes the key performance and structural features of origami-inspired SPRAs reported in the literature.

### 2.7. Other Geometries in SPRAs

In addition to the previously discussed categories, several SPRAs have been developed based on novel geometric configurations and biomimetic design strategies that do not fully conform to the established classifications. These designs often prioritize specific performance objectives, such as enhanced force output, locomotion capability, or compact rotary motion, rather than following a single dominant actuation principle. As a result, they represent an extension of the design space of SPRAs through geometry-driven innovation [96,97].

**Figure 13:**
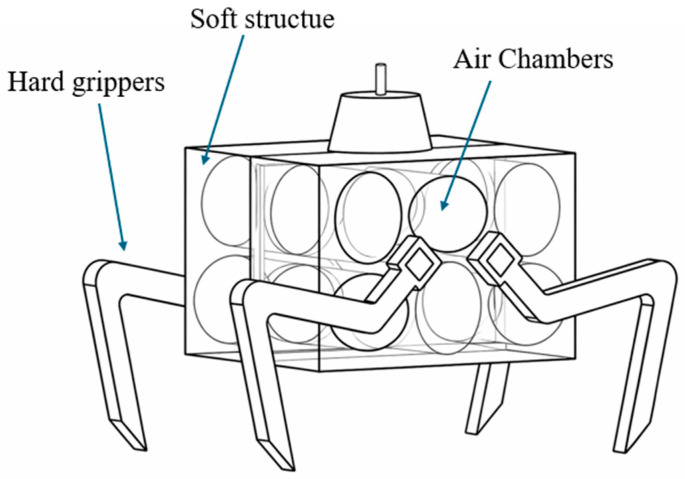
Schematic of soft pneumatic gripper targeted for structural optimization. Adapted from [98].

A common characteristic of these actuators is the use of parameterized geometry and computational optimization to tailor performance. For example, actuator designs based on optimized air chamber layouts (Figure 13) have been developed using finite element modeling to increase gripping force while maintaining compliance [98]. These approaches demonstrate how geometric tuning alone can significantly influence actuator behavior without fundamentally altering the actuation mechanism.

Another notable approach focuses on achieving continuous rotary motion through system-level integration. A lightweight servo-type pneumatic rotary actuator has been developed using an array of nine linear bellows actuators driving a central mechanism, achieving continuous rotation with encoder-based feedback and torque output of approximately 0.53 Nm at 1 bar [99]. This demonstrates that complex rotary functionality can be achieved by combining multiple simple actuation units within a coordinated architecture.

SPRAs based on nonstandard geometries demonstrate that actuator performance can be significantly enhanced through innovative structural design and biomimetic inspiration. These systems expand the functional capabilities of soft pneumatic actuators by enabling specialized behaviors such as continuous rotation and optimized force generation. However, a key limitation of this category is the lack of a unified design principle, which makes systematic comparison and generalization difficult. Many of these designs are highly application-specific, limiting their scalability and transferability to other use cases. In addition, the reliance on complex geometries or system-level integration often increases fabrication complexity and control requirements. While geometry-driven optimization offers powerful design flexibility, it can also lead to solutions that are difficult to standardize or reproduce. Consequently, these actuators are best viewed as exploratory or application-specific designs, rather than a cohesive category, highlighting the need for future work to extract generalizable design principles from these diverse approaches. Table 7 summarizes the key performance and structural features of SPRAs with other geometrical configurations reported in the literature.

**Figure 14:**
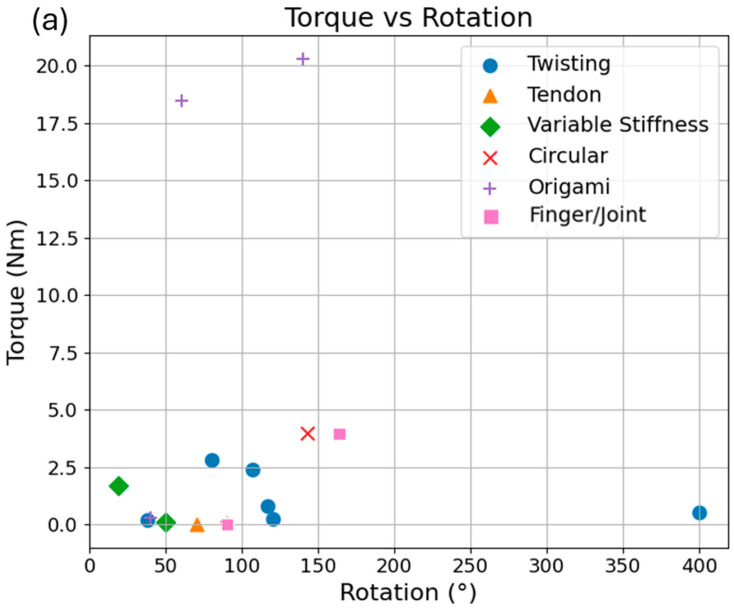

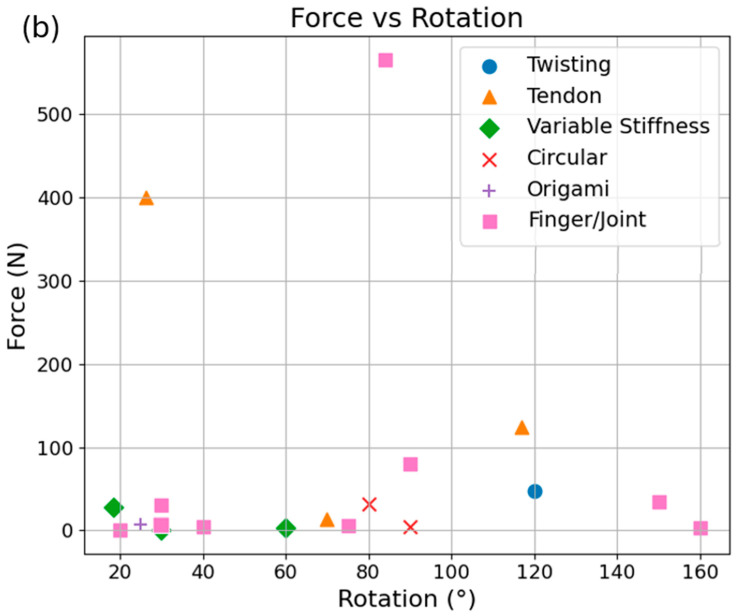
Comparison of (**a**) torque and (**b**) force as a function of rotational range across different categories of SPRAs. Only actuators with available torque or force data are included in the corresponding plots. Marker styles denote actuator categories.

To enable a unified comparison of actuator performance, scatter plots of (a) torque and (b) force versus rotational range are presented in Figure 14. Due to inconsistencies in the literature, only actuators with available torque or force data are included in the respective plots, while systems reporting incomplete or non-standard metrics are excluded. This approach ensures a consistent basis for comparison across different SPRA categories. The torque–rotation plot reveals that origami-inspired SPRAs achieve the highest torque outputs, even at moderate rotational ranges, indicating their strong load-bearing capability. In contrast, twisting actuators exhibit a broader distribution in rotational range, including very large deformations, but generally produce moderate torque levels. Circular actuators provide relatively controlled rotational motion with moderate-to-high torque, while variable-stiffness designs span a wide range of torque outputs depending on their hybrid structural configurations. Tendon-assisted actuators, on the other hand, tend to generate comparatively low torque, reflecting their emphasis on force transmission and compact actuation.

The force–rotation plot complements this analysis by showing that tendon-assisted and finger/joint-inspired actuators dominate in force output, reaching significantly higher force levels compared to other categories. Twisting and variable-stiffness actuators typically produce moderate forces, whereas origami and circular designs tend to operate at lower force levels despite their torque capabilities. Overall, these trends highlight the fundamental trade-offs between torque generation, force output, rotational range, and structural complexity across different SPRA architectures. No single actuator type simultaneously maximizes all performance metrics, emphasizing the importance of selecting actuator designs based on specific application requirements.

## 3. Research Gaps and Future Outlook

**Figure 15:**
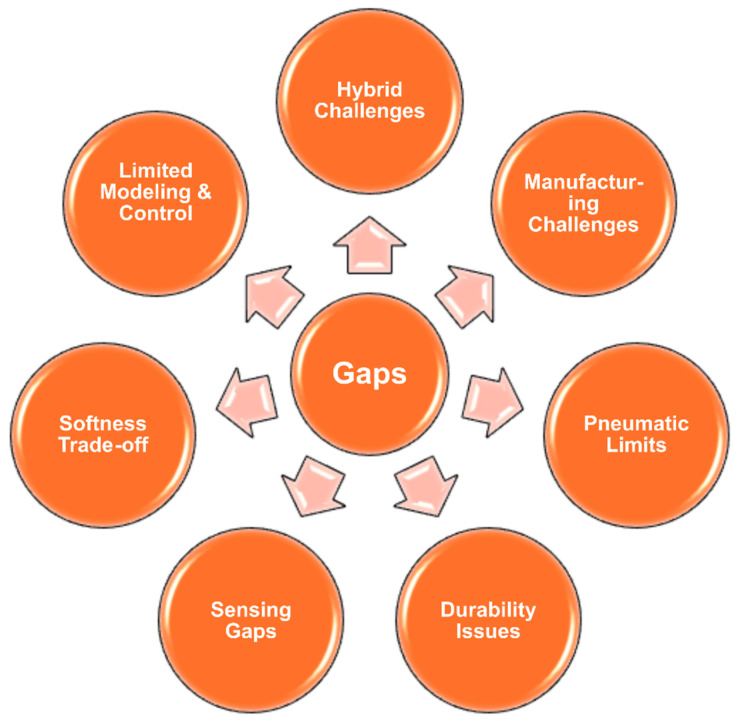
Research gaps and challenges in SPRAs.

Research on SPRAs still faces several critical gaps that shape their future outlook (Figure 15). One of the main challenges is the limited understanding and modeling of large deformation behavior in soft pneumatic actuators. Accurate modeling of these systems remains challenging due to the need to couple hyperelastic material models (e.g., Yeoh or Ogden models) with fluid–structure interaction effects under large deformation [100]. Furthermore, the inherent trade-off between softness and force output continues to hinder progress. Soft materials enable safe, adaptive interaction but generally lack the rigidity required for high-torque or load-bearing tasks [101,102]. Addressing this balance demands exploration of new materials or hybrid soft–hard design strategies that maintain flexibility without sacrificing actuation performance [31,103]. Embedded sensing technologies also remain underdeveloped; integrating compact, robust sensors within actuator structures for real-time monitoring of deformation and environmental interaction is still in its infancy, yet it is crucial for closed-loop control and autonomy [104,105,106]. Another pressing issue is durability and fatigue resistance, as repetitive large deformations or rotational cycles often cause material fatigue and failure, highlighting the need for improved materials and designs that enhance longevity and damage tolerance [107]. Additionally, pneumatic supply and control infrastructure, including portable compressors and pumps, limits actuator autonomy and deployment, calling for innovative research into efficient, compact pneumatic sources or alternative actuation principles [108,109]. Although advanced manufacturing techniques such as 3D printing and multimaterial fabrication have improved actuator development, they still encounter challenges in precision, repeatability, and functional integration, particularly at miniature scales [110,111]. Finally, while hybrid integration of soft actuators with rigid components and electronics shows great potential, it necessitates new design frameworks and material compatibility studies to achieve seamless mechanical and functional synergy [112,113]. Collectively, these research gaps present fertile ground for advancing performance, durability, and real-world adoption of SPRAs across a wide range of robotic and industrial applications.

SPRAs are increasingly positioned to play a pivotal role in sectors where sustainable, lightweight, and inherently safe automation is essential. Recent advances in 3D-printable, scalable, and multi-functional actuator designs are driving down production costs and fostering broader adoption across varied domains. Progressive research in novel materials, advanced control strategies, and hybrid integrations continues to elevate performance in industrial, medical, and consumer applications [114,115].

As robotic systems become more deeply woven into daily life, it remains crucial to innovate in soft actuator design for enhanced sustainability and operational durability, ensuring long-term reliability without loss of structural functionality [116]. The development of embedded sensing technologies is a critical next step, offering actuators expanded functionality and enabling greater adaptability and intelligence in modern robotic platforms [117,118].

A major transformative development is the adoption of advanced manufacturing methods, particularly 3D printing, which significantly improves accessibility and cost-effectiveness for SPRAs. By directly fabricating intricate actuator geometries from digital models, 3D printing reduces manual labor and post-processing costs, streamlining production and enabling rapid prototyping and customization at lower expense. The capacity to use multimaterial printing allows for the integration of reinforcement layers and functional components, further enhancing both the performance and versatility of these devices. The generation of hybrid soft–hard systems can represent a revolutionary paradigm shift. These hybrid robots can have the combined benefits of hard and soft actuators [119].

## 4. Conclusions

In summary, soft pneumatic rotary actuators (SPRAs) are emerging as a highly promising actuation technology for next-generation soft robotic systems, particularly in applications that demand safe human interaction, lightweight structures, and adaptable motion. Their ability to produce smooth rotational and multi-DOF movements makes them attractive for wearable assistance, medical devices, soft manipulators, and compact robotic joints.

A comprehensive review of the existing literature was conducted. Seven categories were proposed on the basis of design architecture and operating principles. Through this classification, the various approaches in SPRAs can be made more understandable and easier to explain. Each category is characterized by a distinct mechanical or structural approach by which pneumatic pressure is converted into controlled rotational output, so that diverse performance requirements, such as torque capacity, angular displacement, and response speed, can be met.

Despite rapid progress, SPRAs remain limited by several fundamental challenges that must be addressed to enable reliable, high-performance deployment beyond laboratory settings. Key priorities include more accurate modeling of large-deformation, as well as multi-directional expansion under complex loading, along with control frameworks capable of accommodating strongly nonlinear and dynamic material behavior to achieve precise, repeatable motion in practice. Equally important is the persistent trade-off between compliance and force output. This drives the development of novel materials and hybrid soft–hard architectures that maintain safe, adaptive interaction while improving torque capacity and load-bearing capability. Future SPRAs will also rely on advances in embedded sensing for real-time state estimation and closed-loop autonomy, as well as improved durability and fatigue resistance under repeated rotational cycling. In addition, autonomy is constrained by the bulk and inefficiency of pneumatic supply and control hardware, highlighting the need for compact, energy-efficient pressure sources or alternative actuation concepts. Finally, although advanced manufacturing, particularly multi-material 3D printing, has already increased design complexity and accessibility, challenges in precision, repeatability, and functional integration at smaller scales persist. Resolving these issues, together with robust material-compatibility and system-level design frameworks for hybrid integration, will be decisive in translating SPRAs into sustainable, lightweight, and inherently safe automation solutions across industrial, medical, and consumer robotics.

## Figures and Tables

**Table 1:** Key performance and structural features of twisting SPRAs.

Actuator	Rotation (°)	Torque (Nm)	Force (N)	Pressure (kPa)	Description	Notes
Fiber-reinforced [19]	~80	~2.8	N/A	5–25	Elastomeric chamber + fibers	Positive pressure
Wire-reinforced [34]	24	N/A	N/A	0–50	Actuator + SMA wires	Hybrid actuation
Pre-twisted tube [35]	±107	2.4	N/A	0–150	Hybrid soft–hard structure	Bidirectional
Extension rotary [33]	400	0.5	N/A	0–500	3 actuators around tube	Large rotation
Vacuum-powered [36]	120	~0.25	~47	−70–0	Seamless chamber	Negative pressure
Freeform chamber [37]	116.7	0.81	N/A	−100–70	Monolithic structure	Bidirectional
Twisting muscle [38]	N/A	0.0188	N/A	−60–0	Helical collapse design	Torsion per length
Optimized joint [39]	~38	0.2023	N/A	−50–50	Topology optimized	Multi-mode

**Table 2:** Key performance and structural features of finger- and joint-inspired SPRAs.

Actuator	Rotation (°)	Torque (Nm)	Force (N)	Pressure (kPa)	Description	Notes
Detachable actuators [46]	160	0.4	3.5	0–90	Detachable bellows	Force and torque both reported
E-Gripper [49]	150	N/A	35	0–150	Chambers + skeleton	Grip force
Soft hand [50]	90	N/A	N/A	0–180	Multi-joint + feedback	Motion-focused
3-Chamber actuator [51]	20	N/A	0.8	0–70	3 air chambers	N/A
Hybrid gripper [52]	90	N/A	80	0–115	Bellows + rigid constraints	N/A
Spider-joint [53]	30	N/A	8.3	0–120	Double-constrained balloon	Bioinspired
Frog-inspired [54]	90	~0.035	N/A	0–120	Articulated pneumatic	Locomotion-focused
Proprioceptive [55]	84	N/A	564.5/302.4	0–150	Origami + rigid frame	Max + grip force
Spider-leg gripper [56]	75	N/A	5.7/15	0–207	Soft joints + linkages	Lift force included
Hybrid pneumatic [57]	40	N/A	4	0–200	Auxetic chamber + rigid frame	N/A
Reconfigurable modular [20]	30	N/A	30	0–80	Modular balloon system	N/A
Composite finger [43]	50	N/A	N/A	0–10	Tendon + pneumatic	Motion-focused

**Table 3:** Key performance and structural features of tendon assisted SPRAs.

Actuator	Rotation (°)	Torque (Nm)	Force (N)	Pressure (kPa)	Description	Notes
Limb rehabilitation robot [63]	26.4	N/A	400	0–200	Soft pneumatic + tendon	N/A
Multi-joint exosuit [66]	N/A	N/A	N/A	N/A	Tendon-driven hip + rigid exoskeleton	0.2 Nm/kg (normalized torque)
Antagonistic pair [67]	117	N/A	~124	0–110	Tendon-driven, antagonistic	Lift: 2 kg
Compact surgical actuator [68]	70	0.003	13	0–400	Pneumatic body + cam	Small-scale torque

**Table 4:** Key performance and structural features of variable-stiffness SPRAs.

Actuator	Rotation (°)	Torque (Nm)	Force (N)	Pressure (kPa)	Description	Notes
Assistant arm [71]	137	N/A	N/A	0–400	Pneumatic chambers + cables	Variable stiffness via cable-pneumatic coupling
Wave spring [72]	50	0.08	N/A	0–86	Silicone actuators + wave springs	Stiffness via embedded springs
Joint with vertebra [73]	18.5	1.7	27.4	0–90	Rigid core + origami actuators	Antagonistic stiffness control
Hybrid module [74]	30	N/A	0.6	0–200	Soft + rigid + sensing	Modular stiffness tuning
Tactile arm [75]	70	N/A	N/A	−50–0	3D-printed bellows + tendons	Integrated sensing
Helical wrist [78]	32/58	N/A	N/A	0–50	Parallel helical actuators	Twist/bend modes
Hybrid 10 DoF [77]	~48/±42	N/A	N/A	0–80	Bellows + rigid	Multi-axis stiffness
Bistable system [76]	60	N/A	3.4	N/A	Instability-driven modules	Snap-through stiffness

**Table 5:** Key performance and structural features of circular SPRAs.

Actuator	Rotation (°)	Torque (Nm)	Force (N)	Pressure (kPa)	Description	Notes
Rotary actuator [80]	−16 to 90	N/A	4	−45 to 18	Antagonistic silicone actuators on rotary joint	Bidirectional motion
Load-bearing joint [15]	142.9	>4	N/A	0–240	Two semicircular actuators + brake	Braking torque
Thumb assistance [81]	80	N/A	32	0–200	Dual independent actuators	Wearable application

**Table 6:** Key performance and structural features of origami SPRAs.

Actuator	Rotation (°)	Torque (Nm)	Force (N)	Pressure (kPa)	Description	Notes
Dual morphing [90]	130	N/A	N/A	0–1.5	Stretchable origami units	Multifunctional motion
Foldable mechanism [92]	40	0.3	3	0–60	3D spherical origami joints	Multi-base folding
Soft origami [93]	60	18.5	N/A	0–180	Integrated stiffness structure	High torque
Soft robotic wrist brace [94]	25	N/A	7	0–160	Origami actuator for wearable support	Assistive device
Origami joint [95]	140	20.3	N/A	0–50	Hybrid hard–soft rotary structure	Bidirectional rotation

**Table 7:** Key performance and structural features of other geometries of SPRAs.

Actuator	Rotation (°)	Torque (Nm)	Force (N)	Pressure (kPa)	Description	Notes
Optimized gripper [98]	N/A	N/A	N/A	−5 to 4	Optimized chamber layout	Geometry-driven design
Servo actuator [99]	Continuous	0.53	N/A	0–100	Bellows-based rotary system	Continuous rotation

## Data Availability

No new data were created or analyzed in this study. Data sharing is not applicable to this article.
